# Focal Therapy for Prostate Cancer: Rationale and Treatment Opportunities

**DOI:** 10.1016/j.clon.2013.05.002

**Published:** 2013-08

**Authors:** V. Kasivisvanathan, M. Emberton, H.U. Ahmed

**Affiliations:** ∗ Division of Surgery and Interventional Sciences, University College London, UK; † Urology Department, Division of Surgery, University College London Hospitals Trust, London, UK; ‡ NIHR UCLH/UCL Comprehensive Biomedical Research Centre, London, UK

**Keywords:** Focal ablation, focal therapy, future perspective, outcomes, prostate cancer, rationale

## Abstract

Focal therapy is an emerging treatment modality for localised prostate cancer that aims to reduce the morbidity seen with radical therapy, while maintaining cancer control. Focal therapy treatment strategies minimise damage to non-cancerous tissue, with priority given to the sparing of key structures such as the neurovascular bundles, external sphincter, bladder neck and rectum. There are a number of ablative technologies that can deliver energy to destroy cancer cells as part of a focal therapy strategy. The most widely investigated are cryotherapy and high-intensity focussed ultrasound. Existing radical therapies, such as brachytherapy and external beam radiotherapy, also have the potential to be applied in a focal manner. The functional outcomes of focal therapy from several phase I and II trials have been encouraging, with low rates of urinary incontinence and erectile dysfunction. Robust medium- and long-term cancer control outcomes are currently lacking. Controversies in focal therapy remain, notably treatment paradigms based on the index lesion hypothesis, appropriate patient selection for focal therapy and how the efficacy of focal therapy should be assessed. This review articles discusses the current status of focal therapy, highlighting controversies and emerging strategies that can influence treatment outcomes for the future.

## Statement of Search Strategies Used and Sources of Information

The MEDLINE database was searched using the PubMed Portal with the following search terms: ‘(focal and (therapy or radiotherapy or radiation or high-intensity focal ultrasound or laser or photodynamic or electroporation or ferromagnetic or cryotherapy or radiofrequency ablation or brachytherapy or microwave or proton or cyberknife)) and prostate cancer’. The search was expanded by looking through related articles and the references of included papers for further relevant papers.

## Introduction

Focal therapy is an emerging treatment modality for localised prostate cancer that aims to reduce the morbidity seen with radical therapy, while maintaining cancer control. This review summarises the rationale for focal therapy, its current status and future perspectives.

## Rationale for Focal Therapy

Current options for men with localised prostate cancer include active surveillance and radical therapy. The ideal treatment would provide oncological cure with few side-effects. Although radical therapy offers treatment with curative intent there can be a high rate of associated functional complications, with erectile dysfunction seen in 24–90%, urinary incontinence in 2–72% and rectal toxicity in 2–15% [1–4]. These complications detrimentally affect quality of life [2,5].

In the era of prostate-specific antigen (PSA) screening, there has been an increase in the detection of prostate cancer [6]. Men are being diagnosed at an earlier stage and the proportion of men with low-risk disease is increasing [7,8]. The debate over population-based PSA screening continues, with differing findings from the European Randomised Study of Screening for Prostate Cancer (ERSPC) and the Prostate, Lung, Colorectal and Ovarian (PLCO) randomised controlled trials leading to the recent US Preventive Services Task Force recommendation against population-based PSA screening [9,10]. However, the high rates of contamination of the control arms in ERSPC Rotterdam Section (31%) and the PCLO trial (40–52%) [10,11] and the emerging patterns of PSA screening in other countries [12] show that physicians and men favour PSA screening. These men may be unnecessarily exposed to the harms of radical treatment. The Prostate cancer Intervention Versus Observation Trial (PIVOT) study, which randomised men diagnosed during the early PSA screening era between watchful waiting and radical surgery, suggests that men with low-risk localised prostate cancer have no benefit from treatment with regards to prostate cancer-specific mortality [13]. Other minimally invasive options for these men should be actively considered.

Active surveillance takes advantage of the slow progression of low-risk disease, allowing about two-thirds of men who enter an active surveillance programme to avoid radical treatment and its side-effects [14,15]. The mortality rates for patients on active surveillance is low at 0–1%, but due to the inherent errors of the diagnostic transrectal biopsy, it is unclear which cancers are intermediate- and high-risk at baseline and there are concerns that delaying radical therapy may lead to disease progression and missing the opportunity for curative treatment. Furthermore, the anxiety and burden of repeated hospital visits, PSA tests and biopsies to the individual and healthcare systems should not be underestimated [16–19]. This may explain why less than 8% of eligible patients in the USA and 39% of those eligible in the UK opt for active surveillance [7,20].

Focal therapy has been proposed as an alternative minimally invasive technique that aims to destroy the tumour itself or the region containing the tumour in order to preserve surrounding non-cancerous tissue. The goal is to maintain disease control at acceptable levels, while preserving erectile, urinary and rectal function by minimising damage to the neurovascular bundles, external sphincter, bladder neck and rectum. This approach has gained increasing attention over the last 5 years, with encouraging evidence accumulating on functional outcomes and short-term oncological outcomes [21–25].

## Focal Therapy Treatment Strategies

**Fig 1:**
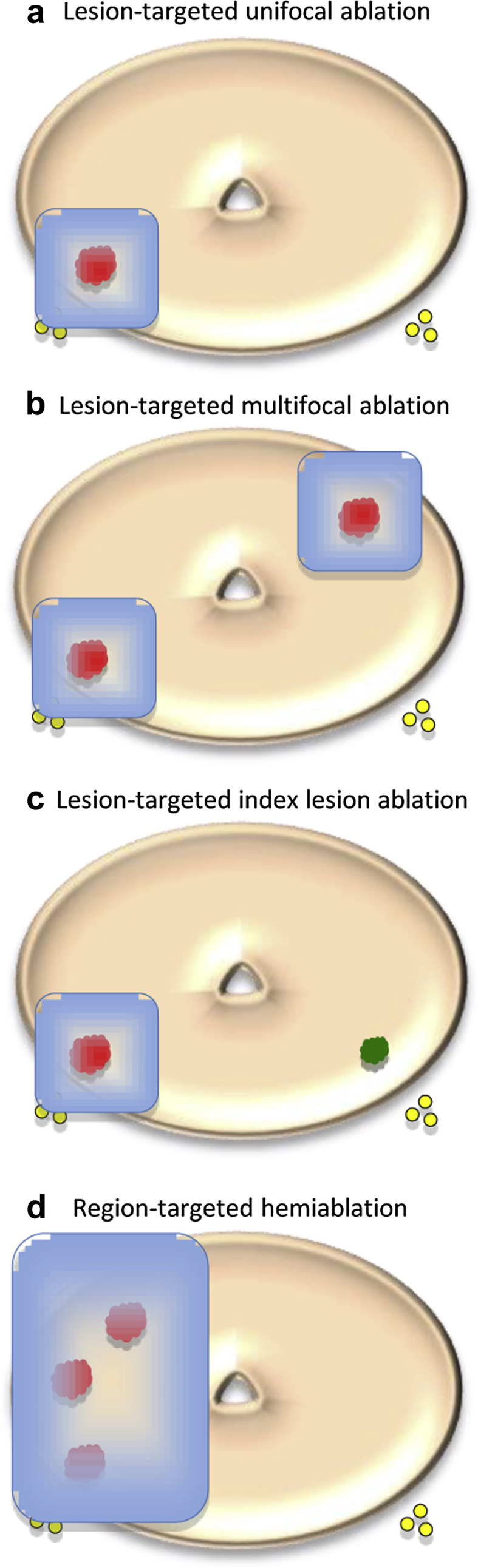
Diagrammatic representation of focal therapy strategies. The red lesion represents clinically significant prostate cancer and the green lesion represents clinically insignificant prostate cancer. The yellow circles represent the neurovascular bundles and the blue rectangle represents the ablation zone. Lesion-targeted therapy is represented by (a)–(c). In (a), unifocal ablation preserves the contralateral neurovascular bundle. In (b), although clinically significant cancer is present bilaterally, one neurovascular bundle is still spared. In (c), clinically insignificant cancer near the second neurovascular bundle is not treated. Only the index lesion is treated, allowing preservation of one neurovascular bundle. In (d), an example of region-targeted therapy, hemi-ablation, is presented.

A number of focal therapy strategies have commonly been used (Figure 1). In general, they differ by whether they attempt to ablate specific cancer foci (lesion-targeted therapy) or whole regions containing cancer (region-targeted therapy).

Focal therapy is classically considered for men with a single discrete tumour or several foci in one half of the prostate. A recent systematic review showed that 13–67% of patients have unifocal or unilateral disease [26]. A further strategy, which extends the proportion of men eligible for focal therapy treatment, is the index lesion ablation strategy. This involves treating only the largest and highest-grade tumour (the index lesion), while sparing small foci less than 0.5 ml, with a Gleason Score less than 7, which may not contribute to disease progression over a 10–20 year period.

In the presence of multifocal disease, the index lesion has been shown to determine the clinical progression of disease [26–29]. Molecular genetics studies suggest that a single tumour focus is responsible for metastasis and disease progression and that this focus is the index lesion [30,31]. The critical tumour volume that correlates to a ‘clinically significant’ lesion that will probably contribute to disease progression, has often been proposed as 0.5 ml [32,33].

Region-targeted therapy treats a larger area of the prostate in the region of the tumour with the rationale that treating multiple tumour foci may give a greater certainty of cure while still preserving vital structures [34]. Hemi-ablation of the half of the prostate containing the tumour is the most commonly reported focal therapy strategy. Subtotal prostate ablation of volumes greater than half the prostate, for example hockey stick ablation, has also been shown [35]. However, the more extensive the treatment, the more likely the functional outcomes will approach those of radical therapy. Overall, it is estimated that between one-half and two-thirds of men with localised prostate cancer may be amenable to some form of focal therapy [36,37].

## Patient Selection for Focal Therapy

Most focal therapy trials have included men with low-risk (low volume, Gleason grade 6) disease for whom active surveillance has shown a very low 10–15 year mortality [14]. However, for those groups that include intermediate-risk (Gleason grade 3 + 4) disease in active surveillance protocols [38] or those that consider focal therapy a potential alternative to radical therapy [39], patients with intermediate- and high-risk (Gleason grade 4 + 3 or greater) disease would be suitable and have been included [24,40], although there is much disagreement on this [41]. A limitation of including intermediate- and high-risk men is their higher rate of micro-metastases and disease progression, even after radical therapy [42,43], which means including them in a focal therapy trial may increase the risk of early treatment failure, making it difficult to fully interpret treatment efficacy.

Retreatment of tumours with focal therapy is possible, although the greater the amount of tissue ablated on the first occasion, the more limited the second treatment can be [24]. After focal therapy, men are still eligible for radical therapy, although it may be technically more challenging [44]. Focal salvage therapy after external beam radiotherapy has also been shown [45,46]. This approach is based on the observation that the main site of recurrence is usually at the site of the original index lesion [47–49].

## Localisation of Disease

For focal therapy to be successful, key requirements are the ability to accurately and reliably identify all clinically significant tumours in the prostate, guide the focal ablation energy to the tumour and assess the treated area to determine treatment efficacy. There is no single modality that meets these requirements, although currently a combination of biopsy and imaging techniques is best practice.

For the selection of men for focal therapy, a 5 mm transperineal template prostate mapping (TPM) biopsy is the current recommended standard [50]. It can provide three-dimensional coordinates of specific cancer foci. TPM has an approximate 95% sensitivity and negative predictive value in detecting and ruling-out clinically significant cancer [51,52]. In patients who have had unilateral cancer identified on transrectal ultrasound (TRUS) biopsy, 61% were found to have bilateral cancer after reclassification by TPM, and 23% of patients had Gleason scores upgraded to 7 or higher [53]. The consequences of the random and systematic error of TRUS biopsy is that clinically significant cancers may be missed, men may be inappropriately selected for focal therapy and when assessing treatment efficacy, differentiating between ablation failure or sampling error may be difficult. TPM is not without its own burden, however. It is time-consuming, requires a general anaesthetic and is associated with a urinary retention rate of 2–11% [54–57].

Elastography, tissue characterisation imaging modalities and contrast-enhanced ultrasound have shown variable degrees of success in the identification of clinically significant cancer [58–62], although the imaging modality that has attracted the most interest is multi-parametric magnetic resonance imaging (MP-MRI), which uses functional parameters (dynamic contrast-enhanced, diffusion-weighted or magnetic resonance spectroscopy) and anatomical parameters (T2-weighted imaging) and has shown a sensitivity, specificity and negative predictive value of 86, 94 and 95%, respectively, for the identification of tumours greater than 0.5 ml, when compared against radical prostatectomy specimens [63,64]. Evidence suggests that Apparent diffusion coefficient (ADC) values in diffusion-weighted imaging and metabolite ratios in spectroscopy may correlate to the aggressiveness of the cancer [65–67].

The interpretation of prostate MRI does, however, require experienced uro-radiologists and attempts at standardising MRI conduct and reporting have been made [68,69], with validation of reporting standards [70]. TPM still remains necessary before selecting suitable candidates for focal therapy, although we await the results of the PROMIS trial (NCT01292291), which may shed more light on the validity of MP-MRI in the detection of clinically significant prostate cancer.

## Guiding Focal Therapy

Although the three-dimensional location of the tumour for focal ablation can be attained from TPM and MP-MRI, further challenges include the application of the ablative energy to that specific location, given that the prostate is viewed most often by real-time TRUS. Tumour location on MP-MRI images may be reviewed before TRUS-guided focal ablation (visual registration) or it can be registered to the real-time TRUS images with software platforms (software registration), which allow the MRI images or their contours of prostate and tumours to be overlaid on the TRUS viewing screen. As an alternative to using TRUS, MRI can be used to guide procedures and for real-time monitoring of treatment intensity [71]. However, MRI-compatible equipment is expensive, can be cumbersome to use and may require more time to gain expertise. Despite this, significant progress is being made with the technology [72,73].

## Ablative Technologies for Focal Therapy

The technologies with the most functional and oncological outcome data are high-intensity focussed ultrasound (HIFU) and cryotherapy. There have been no randomised control trials comparing the ablative technologies with each other or with standard of care, although a relatively large focal therapy series of 106 patients using a number of ablative technologies has been reported revealing a major complication rate of less than 2% with 100% continence rates postoperatively [25]. It is clear that prospective outcome data are required before these technologies are used routinely in clinical practice, but the key data currently available shall be presented.

### Focal High-intensity Focussed Ultrasound

HIFU is applied by inserting an ultrasound probe into the rectum, which allows both the prostate to be visualised and energy to be delivered to the prostate. HIFU uses energy with more than 5 W of power applied per cm^2^ at frequencies within the 2.25–4 MHz range, focussing this energy on to an intense point in which the density can be as high as 1500 W/cm^2^. The ultrasound wave is absorbed by tissue and converted to heat, typically above 80 °C, which results in coagulative necrosis. In addition, alternating cycles of compression and rarefaction lead to inertial cavitation, which results in cell necrosis. HIFU is best used in men with prostates that have an anteroposterior diameter of less than 40 mm and without prostatic calcification, although the use of transurethral resection of prostate and cytoreduction can allow larger glands to be treated. Outcomes of key studies of focal HIFU are given in Table 1 [24,25,35,74,75]. Continence rates of 90–100%, potency rates of 89–95% and 6–12 month biopsy-free recurrence rates of 77–92% have been reported after treatment, although not all cancer recurrences were clinically significant.

### Focal Cryotherapy

Cryotherapy causes cellular destruction by freezing tissue to below −30 °C. This is achieved by argon-based cryoprobes, which can be inserted transperineally into prostate tumour typically under TRUS guidance. Freezing is achieved by the Joule–Thompson effect, which results in ice-ball formation at the needle tip, which can be manipulated in size. The size of the ice-ball can be crudely monitored with TRUS during the procedure. The ice crystals cause cell death by disrupting cell membranes, causing cell lysis and disrupting the microvasculature leading to cellular ischaemia. Urethral warming devices and thermocouples for systematic temperature monitoring can be used to minimise collateral damage, although urethral warming could theoretically lead to undertreatment of anteroseptal tumours. Outcomes of key studies of focal cryotherapy are given in Table 2 [25,40,76–79]. Continence rates of 96–100%, potency rates of 71–90% and biopsy-free recurrence rates of 60–94% have been reported. Of note, most of the described recurrences occurred in the untreated area of the prostate.

### Focal Photodynamic Therapy

Focal photodynamic therapy (PDT) involves the administration of a photosensitising agent, which when activated by light within the prostate, causes cellular destruction. The photosensitising agent is commonly administered intravenously and is activated by light from optical fibres inserted, most commonly, transperineally into the desired area of the prostate under TRUS guidance. Activation results in the production of reactive oxidative species, such as the singlet oxygen, which cause direct cellular injury and vascular damage and lead to cell necrosis and apoptosis. As PDT depends on the presence oxygen it may not be effective in hypoxic prostate tumours. Reliable treatment planning is difficult to achieve given the requirement of appropriate levels of oxygen, photosensitiser and light in the tumour. Only a few studies showing feasibility of the technique have been published (Table 3) [25,80–82], although a randomised controlled trial of active surveillance versus hemi- or subtotal PDT therapy has almost completed recruitment (NCT01310894).

### Focal Photothermal Ablation

Focal photothermal ablation involves the thermal destruction of cells by application of laser from optical fibres inserted transperineally into the tumour, most commonly under MRI guidance. Increasingly, the 980 nm diode laser is being used with procedures carried out under MRI guidance, requiring fully compatible MRI equipment. After several minutes of treatment, a 1 cm near-spherical ablation zone is produced and although it can be extended by manipulation of the fibre position, this technology is ideal for smaller discrete tumours. MRI allows real-time temperature monitoring to ensure that the temperatures required to ablate tumour cells are reached and to reduce the risk of collateral damage. Key reports of photothermal ablation are described in Table 4 and show the feasibility of the technique [83–87]. Further data on functional and oncological outcomes are awaited and phase I/II trials (NCT01094665) are in progress.

### Focal Therapy Using Radiation

Established treatments such as brachytherapy using high dose rate iridium-192 or permanent low dose rate seeds, commonly of iodine-125 and palladium-103, can also be applied in a focal manner. Seeds can be placed via small catheters inserted transperineally into the prostate using TRUS guidance. With pretreatment planning it is possible to apply a higher radiation dose to the tumour with a lower dose to the surrounding non-cancerous tissue. Barret *et al.* [25] carried out focal brachytherapy in 12 patients who maintained continence post-procedurally, although international index of erectile function scores decreased from 21 at baseline to 14 after the procedure. Larger focal brachytherapy trials are now underway (NCT01354951).

One group has also applied peripheral zone-targeted and urethral-sparing low dose rate brachytherapy, observing encouraging adverse effect profiles and 5 year biochemical-free survival rates in low-risk patients [88,89]. Focal brachytherapy has also shown good use in the salvage setting after failed whole-gland irradiation, where repeat full doses of radiation would not be feasible [90,91].

A recent consensus meeting gave recommendations on patient selection and technical considerations for focal low dose rate brachytherapy [92]. Of note is the emphasis on pretreatment planning, the use of iodine-125 seeds linked with a low activity, consideration of organs at risk, including the shape of the prostatic urethra, and post-implant dosimetry at 24 h or 4 weeks. It was suggested that further modelling is required for prescription dose recommendations and ideal margin size is currently uncertain.

High dose rate brachytherapy to a partial volume of the prostate has been used as a boost after external beam radiotherapy [93] and several ongoing phase I and II trials are investigating its use as a boost to the dominant intraprostatic lesion (NCT00807820, NCT01605097). External beam radiotherapy itself can be used in a focal manner by targeting radiation delivery to specific areas of the prostate. The CyberKnife™, a device that can be used to deliver stereotactic radiation precisely, has been used to target the peripheral zone [94] and dominant lesions [95].

### Other Ablative Energies

Radiofrequency ablation, applied via electrodes inserted transperineally, can induce thermal damage to the prostate and has been shown to be a feasible focal therapy technology for prostate cancer [96,97] and is under further investigation (NCT01423006). Irreversible electroporation is a promising non-thermal ablation technology that has been applied in a number of other solid organ cancers, showing quick, precise and predictable tissue destruction in high-risk anatomical areas [98,99]. A clinical trial of irreversible electroporation in focal therapy of prostate cancer is underway (NCT01726894). Future prospective ablative technologies include the use of gold and magnetic nanoparticles that can be directly injected into tumours, producing heat upon activation by electromagnetic stimulation [100].

## Evaluating Focal Therapy Treatment

**Fig 2:**
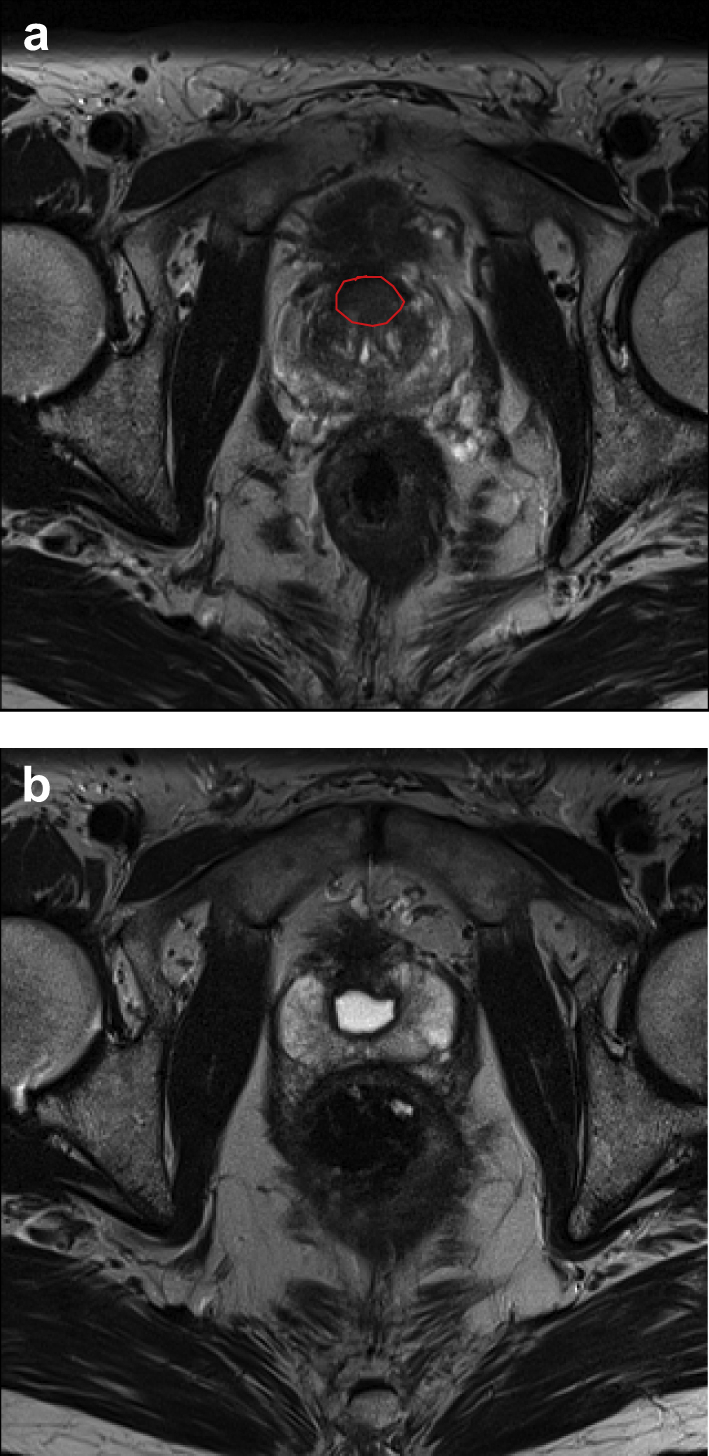
Magnetic resonance imaging (MRI) appearances before and after focal high-intensity focussed ultrasound treatment to the prostate. A T2-weighted prostate MRI image of a man with presenting prostate-specific antigen of 7.7 ng/ml is given in (a). A scanner with a 1.5 Tesla magnet and a pelvic phased array coil was used to capture images. An anterior prostate tumour is indicated by the red circle. Transperineal template prostate biopsies confirmed high volume Gleason 3 + 3 disease. The patient underwent focal high-intensity focussed ultrasound treatment of the tumour. Six months after treatment, the patient underwent repeat MRI and the T2-weighted MRI image obtained is given in (b). The ablation cavity can be seen with no evidence of residual cancer. Prostate-specific antigen at this time was 1.1 ng/ml.

Contrast-enhanced ultrasound and MRI can be used for real-time feedback of tissue destruction with most focal therapy technologies [101,102]. Contrast-enhanced MRI can also be used at an early stage, for example within the first week after focal therapy for verification of treatment effect. Subsequent follow-up for the assessment of oncological control is more challenging. PSA values are difficult to interpret because a variable amount of prostate tissue remains after focal therapy. Further factors influencing post-procedural PSA include the proportion of pre-procedural PSA that was due to the tumour, the efficacy of the ablation therapy and the progression of benign prostate hyperplasia. Thus, a specific threshold nadir for PSA to define biochemical recurrence is unlikely to be derived. Definitions that are currently used to define failure in whole gland radiation therapy (ASTRO [three consecutive rises in PSA from nadir], Phoenix definition [nadir + 2 ng/dl]) have been used in focal therapy, although they have not yet been validated. Certainly, a standardised biopsy scheme to confirm the presence or absence of disease after focal therapy is essential and evidence from whole gland treatment using focal ablation technology supports the role of imaging such as MP-MRI to detect recurrences [103–105]. In summary, a combination of biochemical, histological and imaging results can be used to evaluate the oncological control achieved by focal therapy (Figure 2).

There is a wide range of follow-up protocols in focal therapy trials. In general, PSA is checked 3 monthly for the first year, then 6 monthly [35,74,76,77,106]. A scheduled prostate biopsy is carried out at 6–12 months or with PSA progression.

## Discussion

The aim of focal therapy is to obtain the trifecta status of oncological cure, potency and continence. Treatment strategies can be lesion-targeted or region-targeted with emphasis on preserving neurovascular bundles, bladder neck, external sphincter and rectum. A number of ablative technologies are available, the best studied of which include HIFU and cryotherapy. Although it is clear that more data, particularly on medium- and long-term cancer control, are required, encouraging functional outcomes have now been reported.

Controversies of focal therapy remain. Primarily, current trials do not present medium- and long-term oncological outcome data or comparisons with existing standards of care. There is also no consensus on whether oncological control should be deemed the absence of any cancer or the absence of clinically significant cancer and whether this should be limited to the treated area or include the untreated prostate. The selection of men suitable for focal therapy is another point of contention. Most of those who consider it an alternative to active surveillance would consider low-risk Gleason 6 as suitable, whereas those considering it an alternative to radical therapy would consider higher-risk disease suitable. The index lesion hypothesis has also been challenged, with some weak evidence suggesting that the metastatic deposits may arise from tumours other than the index lesion [107]. Certainly, any untreated lesions must be meticulously monitored in these men.

Much progress has been made in focal therapy over the last 10 years and there is still much to be made. The results of several prospective trials are eagerly awaited, one that will present 3 year cancer control data after HIFU (NCT01194648) and another that will present the results of the index lesion ablation strategy (NCT00988130) (Table 5). If reliable and consistent local control of cancer can be proven, the next step would be randomised controlled trials comparing active surveillance or radical therapy with focal therapy. For these trials to be successful it is important that they are pragmatic and have an adaptive approach to design, for example, the cohort multiple randomised control trial design [108].

Trials need not limit focal therapy to one specific ablative technology, but can include any that have proven efficacy in local cancer control. Indeed, different ablative energies may be advantageous in different circumstances. Outcomes of mortality and metastatic progression would require a large number of men with a 10–15 year follow-up and so other outcomes need to also be considered, such as functional outcomes and side-effects using validated patient questionnaires, the rate of additional systemic therapy and cost-effectiveness. In contrast to the adoption of laparoscopic and robotic radical prostatectomy, with focal therapy we have the opportunity to evaluate the results of a new technique in well-designed prospective clinical trials in a timely manner.

## Conflicts of Interest

M. Emberton and H.U. Ahmed would like to acknowledge funding from the Medical Research Council (UK), the Pelican Cancer Foundation Charity, Prostate Cancer UK, St Peters Trust Charity, Prostate Cancer Research Centre the Wellcome Trust, National Institute of Health Research-Health Technology Assessment Programme, and the US National Institute of Health-National Cancer Institute. M. Emberton receives funding in part from the UK National Institute of Health Research UCLH/UCL Comprehensive Biomedical Research Centre. M. Emberton and H.U. Ahmed receive funding from USHIFU, GSK and Advanced Medical Diagnostics for clinical trials. M. Emberton is a paid consultant to Steba Biotech and USHIFU. Both have previously received consultancy payments from Oncura/GE Healthcare and Steba Biotech. V. Kasivisvanathan receives research grant support from the National Institute of Health Research UK.

## Figures and Tables

**Table 1:** Outcomes of key studies in focal high-intensity focussed ultrasound

Reference	No. patients	Follow-up (years)	Gleason score	PSA (ng/ml)	Disease localisation	Ablation strategy	Continence	Potency	Adverse events	Oncological outcomes
Muto *et al*., 2008 [35]	29	2.6	5–10	5.4	MRI and TRUS biopsy	Posterior hockey stick ablation	29/29 (100%)	NR		1 year biopsy: 13/17 (77%) no cancer 2 years BDFS (ASTRO criteria): 83% in low-risk patients 54% in intermediate-risk patients 0% in high-risk patients
Ahmed *et al*., 2011 [74]	20	1	≤4 + 3	7.3	MP-MRI and TPM	Hemi-ablation	18/20 (90%)	19/20 (95%)	Urethral stricture 1/20 (5%)	6 months biopsy: 17/19 (89%) no cancer 19/19 (100%) no significant cancer
El Fegoun *et al*., 2011 [75]	12	10	≤3 + 4	7.3	TRUS biopsy	Hemi-ablation	12/12 (100%)	NR	Retention 1/12 (8%) UTI 2/12 (17%)	1 year biopsy: 11/12 (92%) no cancer 5 years: Recurrence-free survival – 90% 10 years: Recurrence-free survival – 38% Cancer-specific survival 10/10 (100%)
Ahmed *et al*., 2012 [24]	41	1	≤4 + 3	6.6	MP-MRI and TPM	Lesion-targeted or Region-targeted	38/38 (100%)	31/35 (89%)	Retention 1/41 (2%) UTI 7/41 (17%) Urethral stricture 1/41 (2%) Diarrhoea and urine extravasation 1/41 (2%)	6 months biopsy: 30/39 (77%) no cancer 36/39 (92%) no significant cancer
Barret *et al*., 2012 [25]	21	0.75	6	6	TPM	Hemi-ablation	21/21 (100%)	IIEF-5 decrease from 20 to 14	Retention 5/21 (24%)	NR

PSA, mean/median pre-procedural prostate-specific antigen; TRUS, transrectal ultrasound guided; NR, not reported; BDFS, biochemical disease-free survival; ASTRO criteria, three successive increases in PSA; MP-MRI, multi-parametric magnetic resonance imaging; TPM, transperineal template mapping biopsy; UTI, irinary tract infection; IIEF-5 = International Index of Erectile Function.

**Table 2:** Outcomes of key studies in focal cryotherapy

Reference	No. patients	Follow-up (years)	Gleason score	PSA (ng/ml)	Disease localisation	Ablation strategy	Continence	Potency	Adverse events	Oncological outcomes
Bahn *et al*., 2006 [76]	31	5.8	≤7	4.9	TRUS biopsy	Hemi-ablation	31/31 (100%)	24/27 (89%)		During follow-up: 24/25 (96%) no cancer on biopsy 1/1 recurrence in untreated lobe BDFS (ASTRO)– 26/28 (94%)
Lambert *et al*., 2007 [77]	25	2.3	≤7	6	TRUS biopsy	Hemi-ablation	25/25 (100%)	17/25 (71%)	Retention 1/25 (4%)	During follow-up: 22/25 (88%) no cancer on biopsy 2/3 recurrences in untreated lobe BDFS (Phoenix) –22/25 (88%)
Ellis *et al*., 2007 [78]	60	1.25	≤7	7.2	TRUS biopsy	Hemi-ablation	53/55 (96%)	24/34 (71%)		During follow-up: 21/35 (60%) no cancer on biopsy 13/14 recurrences in untreated lobe BDFS (ASTRO) –48/60 (80%)
Onik *et al*., 2008 [79]	48	4.5	NR	7.8	TPM	Lesion-targeted	48/48 (100%)	36/40 (90%)	Sloughed tissue 1/48 (2%) requiring TURP	During follow-up: 43/48 (90%) no cancer on biopsy 5/5 recurrences in untreated area BDFS (ASTRO) – 45/48 (94%)
Bahn *et al*., 2012 [40]	73	3.7	≤7	5.4	TRUS biopsy	Hemi-ablation	70/70 (100%)	86%		During follow-up: 36/48 (75%) no cancer on biopsy 11/12 recurrences in untreated lobe
Barret *et al*., 2012 [25]	50	0.75	6	6.2	TPM	Hemi-ablation	50/50 (100%)	IIEF-5 decrease from 19 to 14	Retention 4/50 (8%). Gross haematuria 1/50 (2%) requiring irrigation and blood transfusion. 1/50 (2%) stricture. 1/50 (2%) perineal abscess with rectal fistula requiring excision and temporary colostomy.	NR

PSA, mean/median pre-procedural prostate-specific antigen; TRUS, transrectal ultrasound guided; BDFS, biochemical disease-free survival; ASTRO criteria, three successive increases in PSA; Phoenix criteria, PSA nadir + 2 ng/dl; TPM, transperineal template mapping biopsy; TURP, transurethral resection of prostate; NR, not reported; IIEF-5 = International Index of Erectile Function.

**Table 3:** Key studies of focal photodynamic therapy

Reference	No. patients	Gleason score	Photosensitiser	Ablation strategy	Light delivery	Continence	Potency	Adverse events	Oncological outcomes
Windahl *et al*., 1990 [80]	2	NR	Haematoporphyrin derivative (*n* = 1) Photofrin (*n* = 1)	Post-TURP remnant	TU	NR	NR		At 3 months: 2/2 no cancer on control biopsies Reduction in mean PSA from 8 to 1.35 ng/ml
Zaak *et al*., 2003 [81]	6	5–8	5-ALA	Variable	RP (*n* = 1) TU (*n* = 3) TP (*n* = 2)	6/6 (100%)	NR		For one patient who had radical prostatectomy: Necrosis at site of fibre insertion At 6 weeks: Reduction of PSA by 20–70%
Moore *et al*., 2006 [82]	6	3 + 3	Temoporfin	Hemi-ablation	TP	NR	2/3 (67%)	Retreatment (*n* = 4) due to residual cancer Sepsis (*n* = 1) Voiding symptoms requiring recatheterisation (*n* = 2)	At 2 months: 0/6 no cancer on biopsy PSA reduction after 8/10 treatments
Barret *et al*., 2012 [25]	23	6	Padeliporfin	Region-targeted		23/23 (100%)	IIEF-5 decrease from 23 to 13		NR

TURP, transurethral resection of prostate ; TU, transurethral; RP, during radical prostatectomy; TP, transperineal; NR, not reported; IIEF-5, International Index of Erectile Function; PSA, prostate-specific antigen.

**Table 4:** Key reports of focal photothermal ablation

Reference	No. patients	Laser	Ablation strategy	No. fibres	Real-time imaging	Adverse events	Outcomes
Amin *et al*., 1993 [83]	1	805 nm Diomed diode laser	Lesion-targeted	3	US and CT	Mild dysuria	10 days: Biopsy – necrosis in ablation zone
Linder *et al*., 2009 [84]	12	830 nm Indigo diode laser	Lesion-targeted	1–2	3D-US CEUS	Retention (*n* = 2) Perineal discomfort (*n* = 3) Mild haematuria (*n* = 2) Haematospermia (*n* = 2) Fatigue (*n* = 1)	6 month biopsy: 6/12 (50%) no cancer 4/6 recurrences in ablation zone 6 month functional outcome: Potency – 100% of men potent pre-procedure retained potency Continence – no significant worsening of IPSS score
Linder *et al*., 2010 [85]	4	980 nm Visualise diode laser	Lesion-targeted	2–3	CEUS		Good correlation between ablation volume on MRI and ablation volume on H&E stained pathology images
Raz *et al*., 2010 [86]	2	980 nm Visualise diode laser	Lesion-targeted	≥2	3D 1.5 T MRI CEUS		Immediate repeat treatment with new fibre position due to residual vascularised target tissue
Linder *et al*., 2011 [87]	2	980 nm Visualise diode laser	Lesion-targeted	NR	3D robotic 1.5 T MRI		No significant change in IIEF-5 or IPSS scores after treatment

US, ultrasound; CT, computed tomography; MRI, magnetic resonance imaging; CEUS, contrast-enhanced ultrasound; 3D, three-dimensional; H&E, haematoxylin and eosin; NR, not reported; IIEF-5, International Index of Erectile Function; IPSS, International Prostate Symptom Score.

**Table 5:** Notable ongoing trials in focal therapy

Trial number identifier	Focal ablation modality	Phase	Description	Intended no. patients	Selection criteria	Key outcomes
NCT01194648	HIFU	Phase II	Multi-centre Single-arm	272	T1–T3a Gleason ≤ 4 + 3 PSA < 15 ng/ml	Proportion of men free of any cancer and free of clinically significant prostate cancer at 36 months on TPM
NCT00988130	HIFU	Phase II	Single-centre Single-arm Index lesion ablation	26	≤T3b Gleason ≤ 8 PSA ≤ 20 ng/ml	Side-effects and quality of life Absence of cancer in treated area at 12 months by TRUS biopsy
NCT01310894	PDT	Phase III	Multi-centre RCT Active surveillance versus focal therapy	400	≤T2c Gleason ≤ 3 + 3 PSA ≤ 10 mg/ml	Rate of absence of definite cancer at 24 months Rate of failure with observed progression of disease from low risk to higher risk
NCT01094665	Photothermal	Phase I/II	Single-centre Single-arm	60	T1–T2a PSA < 15 ng/ml	Absence of cancer at 4 months on TRUS biopsy
NCT01354951	LDR-brachytherapy	Phase II	Single-centre Single-arm	80	T1c-T2a Gleason 7 in two cores or less PSA < 10 ng/ml	Toxicity at 6 months to 2 years Absence of cancer at 12 and 24 months on biopsy
NCT00807820	HDR-brachytherapy	Phase I	Single-centre Single-arm Selective boost to DIL	56	T2a–2b, Gleason 2–6, PSA 10–20 ng/ml or T3a–3b, Gleason 2–6, PSA ≤ 20 ng/ml or T2a–3b, Gleason 7–10, PSA ≤ 20 ng/ml	Rate of ≥ grade 3 genitourinary or gastrointestinal toxicity at 12 months
NCT01423006	RFA	Phase I	Single-centre Single-arm	7	T1c Gleason ≤ 6 PSA < 10 ng/ml	Absence of cancer at 6 months on biopsy
NCT01726894	IRE	Phase I	Single-centre Single-arm	20	T1–T2c Gleason ≤ 7 PSA ≤ 15 ng/ml	Adverse events at 12 months

HIFU, high-intensity focussed ultrasound; PDT, photodynamic therapy; LDR, low dose rate; HDR, high dose rate; RFA, radiofrequency ablation; IRE, irreversible electroporation; RCT, randomised controlled trial; DIL, dominant intra-prostatic lesion; TPM, transperineal template mapping prostate biopsy; TRUS, transrectal ultrasound; PSA, prostate-specific antigen.
